# Design and application of a point-of-care testing system for triple detection of SARS-CoV-2, influenza A, and influenza B

**DOI:** 10.3389/fbioe.2024.1378709

**Published:** 2024-04-17

**Authors:** Huan Yang, Xiaoming Zhang, Yating Li, Jing Deng, Zhongming Liu, Qiyue Chen, Haiyan Zhang

**Affiliations:** ^1^ Guangzhou University of Chinese Medicine, Guangzhou, China; ^2^ General Hospital of Southern Theater Command, Guangzhou, China; ^3^ Beijing Genome Technology Co., Ltd., Beijing, China

**Keywords:** POCT, smartphone platform, SARS-CoV-2, influenza, PCR

## Abstract

To mitigate the continued impact of SARS-CoV-2, influenza A, and influenza B viruses on human health, a smartphone-based point-of-care testing (POCT) system was designed to detect respiratory pathogens through a nucleic acid test. This compact, light-weight, highly automated, and universal system enables the differential diagnosis of SARS-CoV-2, influenza A, and influenza B in approximately 30 min in a single-tube reaction. Numerous hospitals and disease control and prevention center assessed the triple POCT system’s detection threshold, sensitivity, specificity, and stability, and have concluded that all the assessments were comparable to those of fluorescent quantitative polymerase chain reaction (PCR)-based testing. The triple POCT system is suitable as an onsite rapid-diagnosis device, as well as for pathogen screening at airports and customs.

## 1 Introduction

Coronavirus disease 2019 (COVID-19) caused by the severe acute respiratory syndrome coronavirus 2 (SARS-CoV-2) virus rapidly evolved into a pandemic, severely affecting human health and substantially straining health resources (Grubaugh et al., 2021; Hu et al., 2021; Synowiec et al., 2021). Meanwhile, the influenza A virus (IAV), influenza B virus (IBV), respiratory syncytial virus, and parainfluenza virus have continued to adversely affect human health (Griffiths et al., 2017; Hutchinson, 2018; Kalil and Thomas, 2019; Cools et al., 2021). Transmission prevention and timely treatment relies on the prompt and accurate onsite detection of SARS-CoV-2 and other acute respiratory pathogens. The current gold standard for molecular diagnosis is nucleic acid amplification by polymerase chain reaction (PCR), which is highly sensitive and accurate, but requires trained professional technical staff, expensive laboratory equipment, and long processing times. Thus, PCR tests are not suitable for rapid onsite screening and do not readily facilitate the prompt quarantine and treatment of pathogen carriers (Green and Sambrook, 2019; Jin et al., 2020; Safiabadi Tali et al., 2021; Yüce et al., 2021). Rapid antigen-based testing techniques have seen widespread commercialization, and although highly convenient and rapid, they do not achieve sufficient sensitivity and can lead to false negative results (Fouzas, 2021; Peeling et al., 2022). Numerous studies have investigated the application of loop-mediated isothermal amplification (LAMP) (Li et al., 2021; Ye et al., 2022), recombinase polymerase amplification (RPA) (Woo et al., 2021), and digital PCR(Choi et al., 2022) techniques for the detection of multiple infectious respiratory pathogens. However, these methods are often characterized by limitations such as suboptimal sensitivity or the need for specialized laboratory settings, thus limiting their feasibility for rapid on-site detection (Zhang et al., 2019; Salipante and Jerome, 2020).

Thus, there is an urgent need to develop a highly sensitive, highly specific, and rapid test for concurrent testing of multiple pathogens. This study focuses on the design of a highly sensitive and specific rapid nucleic acid test for a concurrent detection of COVID-19, influenza A, and influenza B. To achieve this, we integrated a sample extraction and enrichment module, an ultrafast thermal cycling PCR module, and a smartphone data processing module to engineer the triple POCT system. The system’s performance and clinical applications were assessed and described as follows.

## 2 Design and components

**FIGURE 1:**
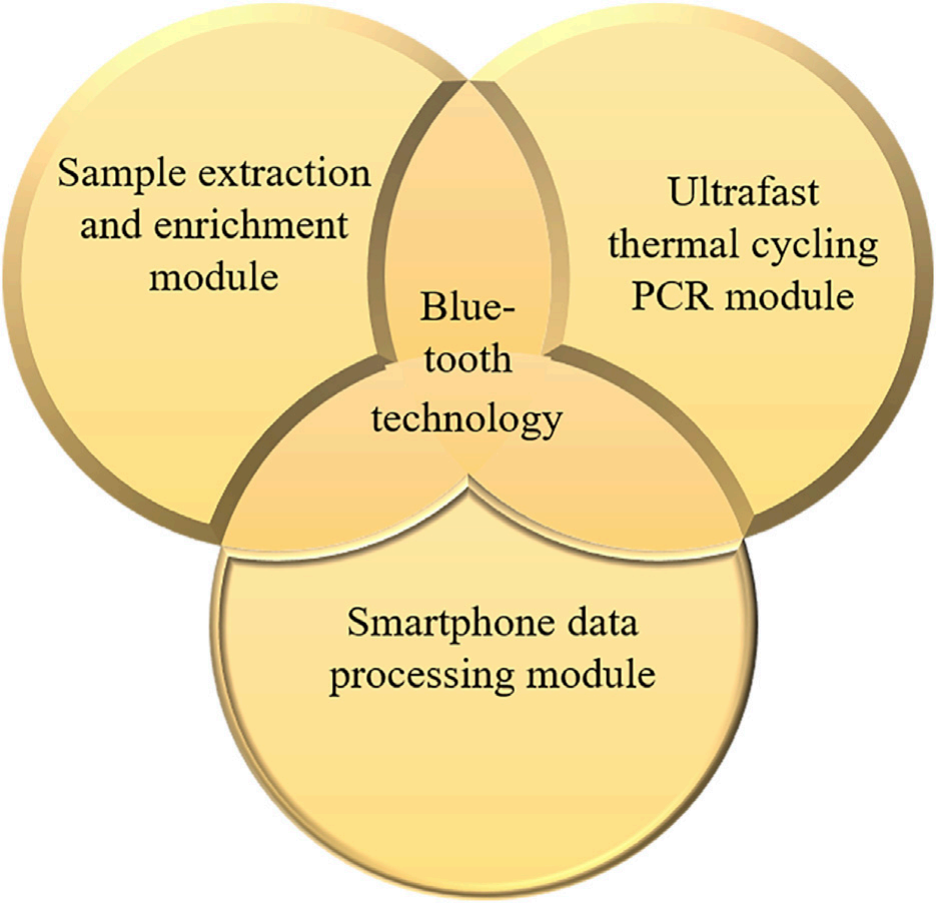
Structure and composition of the COVID-19, influenza A, and influenza B triple POCT system.

The COVID-19, influenza A, and influenza B triple POCT system consists of a sample extraction and enrichment module, an ultrafast thermal cycling PCR module, and a smartphone data processing module, as shown in Figure 1.

### 2.1 Sample extraction and enrichment module

The sample extraction and enrichment module is fundamental to the high sensitivity and specificity of the respiratory pathogen POCT system. The goal of this module is primarily to increase the nucleic acid extraction efficiency, minimize impurities, shorten preparation time, and decrease the required volume of the sample. A high-purity nucleic acid solution is obtained after magnetic sample lysis, nucleic acids are bound to magnetic beas, and washing of nonspecific binders. The sample extraction and enrichment module consists of three components, namely, the extraction and enrichment execution unit, control circuit unit, and 20-well extraction reagent plate unit.

**FIGURE 2:**
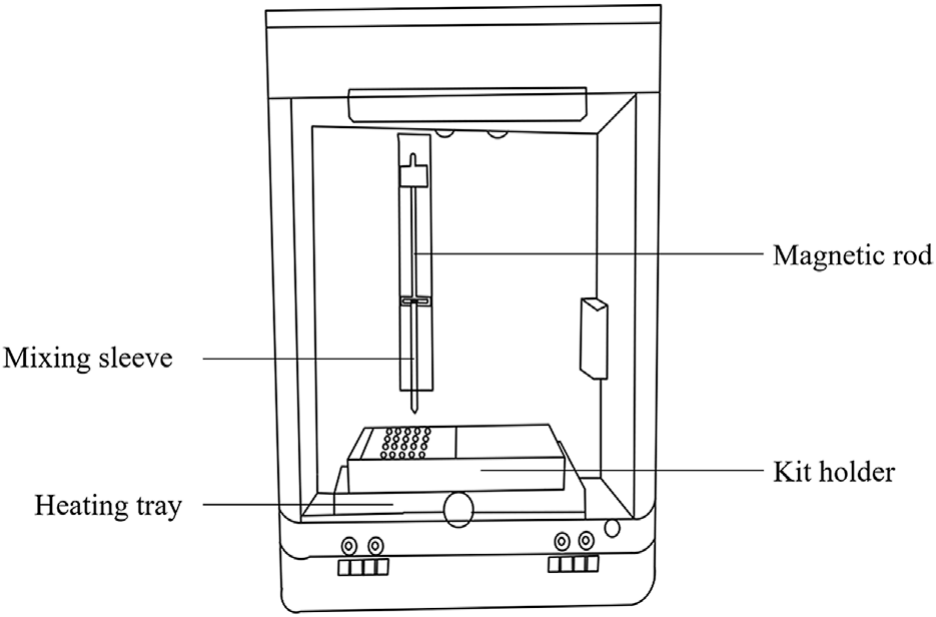
Schematic diagram of the extraction and enrichment execution unit.

The extraction and enrichment execution unit (Figure 2) consists of a magnetic rod, mixing sleeve, and heating tray. The function of this unit is to enable sufficient stirring, maintain a suitable temperature, precise transfer of magnetic beads for sample lysis, magnetic bead adsorption, magnetic bead cleaning, and nucleic acid dissociation. The mixing sleeve is made of smooth, corrosion-resistant polypropylene, with low specific gravity and no nucleic acid binding. Mixing speed and magnitude are controlled by a preset program to achieve sufficient stirring while preventing splashes.

**FIGURE 3:**
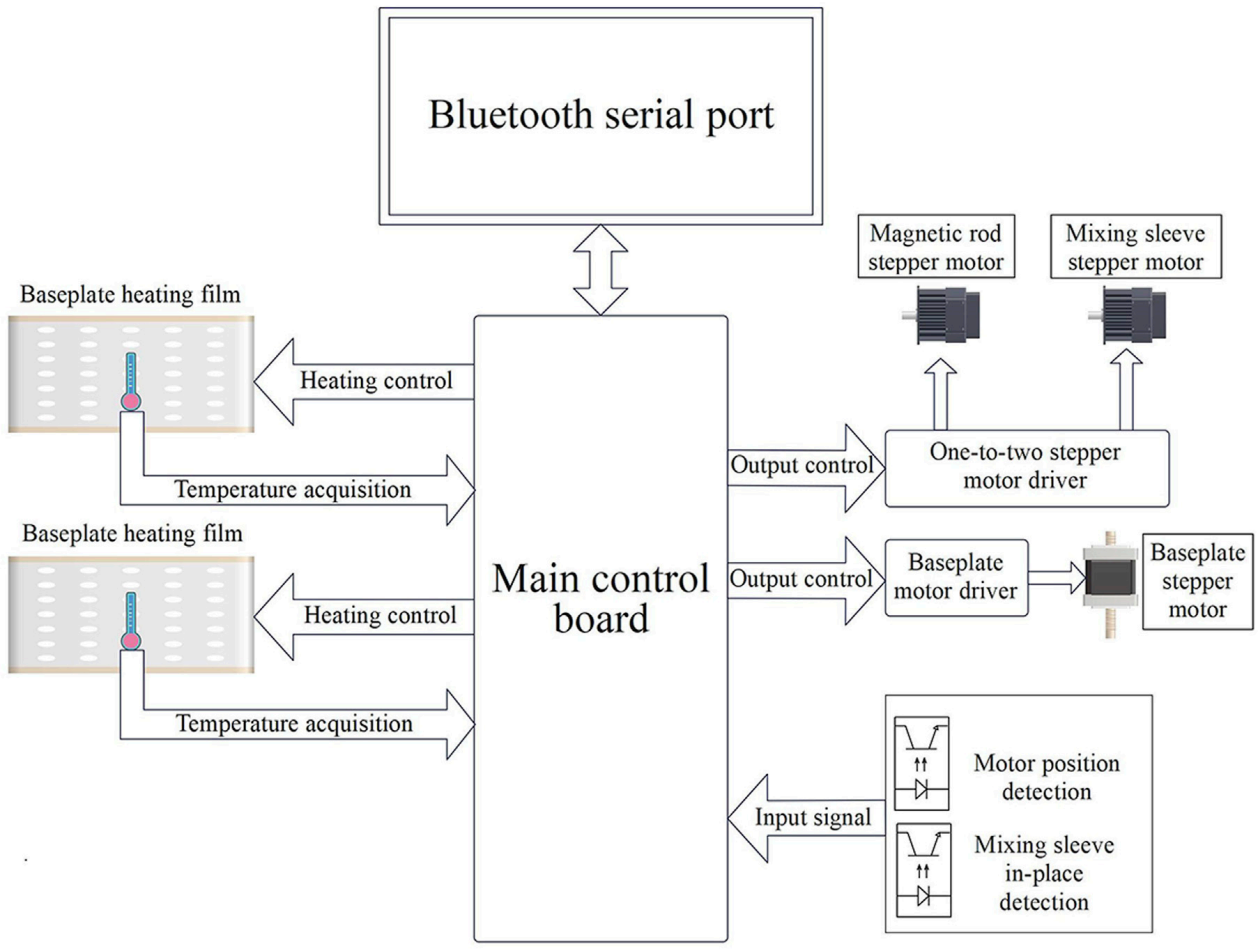
Schematic diagram of the control circuit unit.

The baseplate heating film of the control circuit unit (Figure 3) and its corresponding control circuit provide a suitable and stable temperature for sample lysis, magnetic bead binding, washing, and dissociation. Sufficient stirring and magnetic bead transfer is achieved through the detection of the motor position and mixing sleeve in-position driven by the one-to-two stepper motor.

**FIGURE 4:**
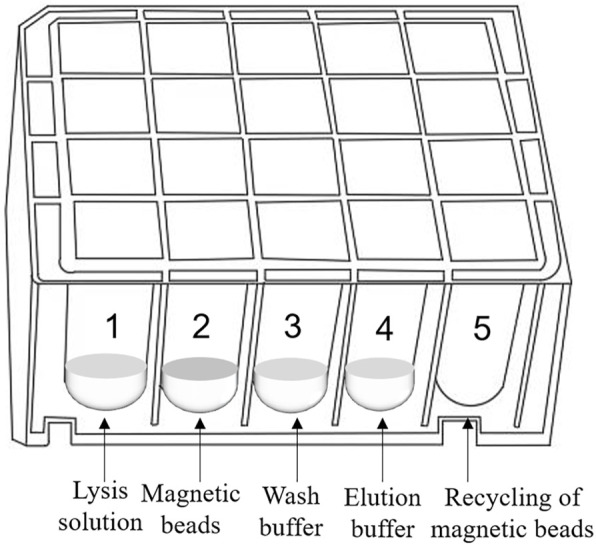
Schematic diagram of the 20-well extraction reagent plate unit.

The 20-well extraction reagent plate unit can concurrently extract and enrich four samples and is compatible with the ultrafast thermal cycling PCR module. Figure 4 shows the extraction reagent layout. Magnetic silica microspheres with super-paramagnetism and biocompatibility are prepared using embedding and interface deposition methods. By evaluating the adsorption performance of magnetic nanoparticles with different particle sizes and surface modifications on nucleic acid molecules, magnetic microspheres composed of polymer nanomaterials are developed. These microspheres have a large surface area, rapid magnetic response, strong adsorption ability, easy elution, and effective ability to achieve sample nucleic acid concentration and purification. The development of proprietary wash and elution buffers ensures that nucleic acid extraction and enrichment of four samples can be completed within 5 min, creating conditions for obtaining a high-purity nucleic acid extraction solution.

### 2.2 Ultrafast thermal cycling PCR module

The ultrafast thermal cycling PCR module consists of a flat reaction cup, an ultrafast thermal cycler designed around the flat reaction cup, a fixed light path fluorescence detection unit, and a lyophilization reagent unit.

**FIGURE 5:**
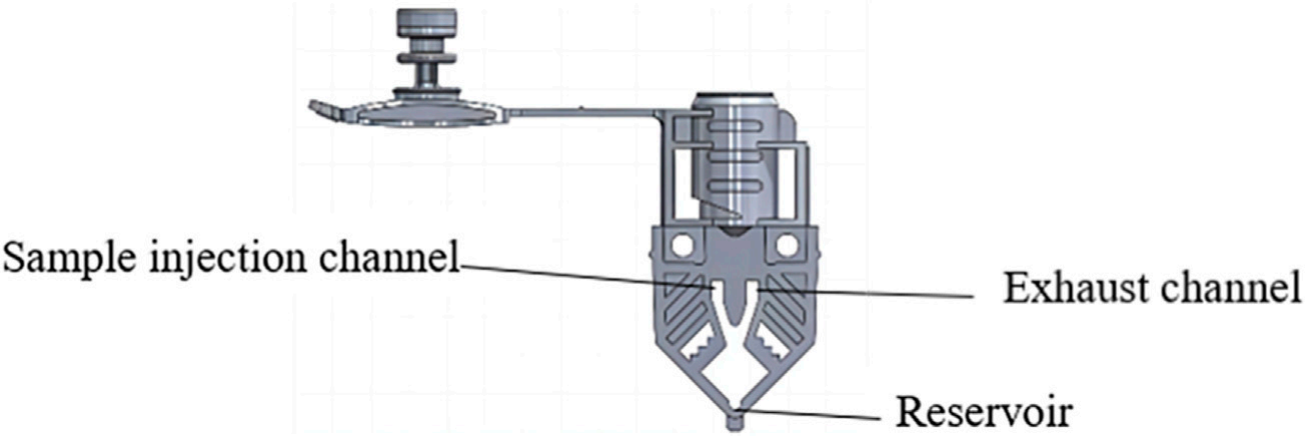
Schematic diagram of the reaction cup.

The flat reaction cup is constructed through high-precision injection molding. The cup contains a sample injection channel and an exhaust channel to expel air bubbles. To ensure that air bubbles are successfully expelled during centrifugation, a reservoir is present at the bottom of the reaction cup, which also aims to decrease the effect of precipitates on the accuracy of fluorescence detection (Figure 5). The reaction cup has a thickness of 0.7 mm, and its shape closely matches that of the heating chip, aiming to increase heat transfer efficiency by maintaining close contact.

**FIGURE 6:**
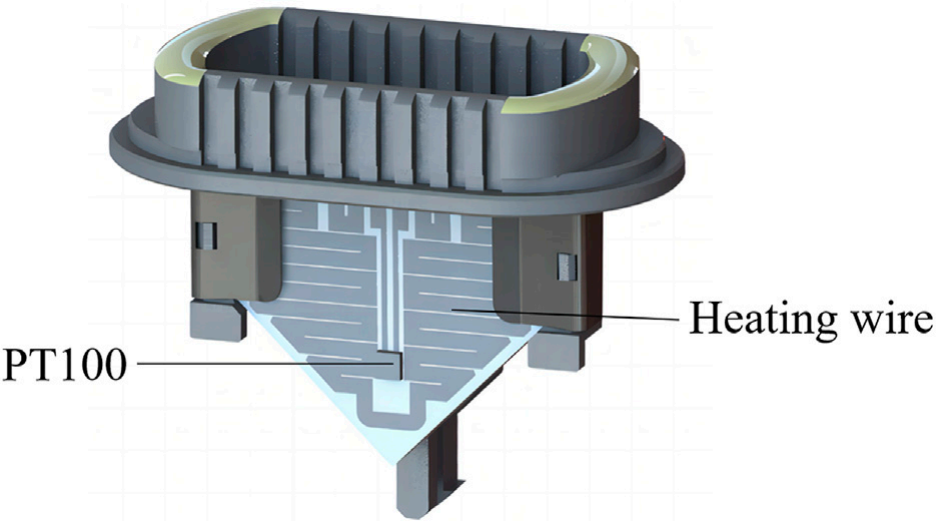
Schematic diagram of the structure of the sintered ceramic heating sheet.

The ultrafast thermal cycling unit consists of a heating unit and a cooling unit. The base material of the heating unit uses conventional ceramic sheets, with the heating wire made of an Ag/Pb alloy. The heating wire, using a unique arrangement, is manufactured from liquid metal coating and sintering to cover the ceramic sheets (hereinafter known as heating sheet). PT100 was selected as the temperature sensor, and a high-temperature epoxy resin glue is used to glue it to the surface of the heating sheet (Figure 6). This miniaturized heating design thus overcomes challenges associated with low power conversion efficiency in traditional heating components.

**FIGURE 7:**
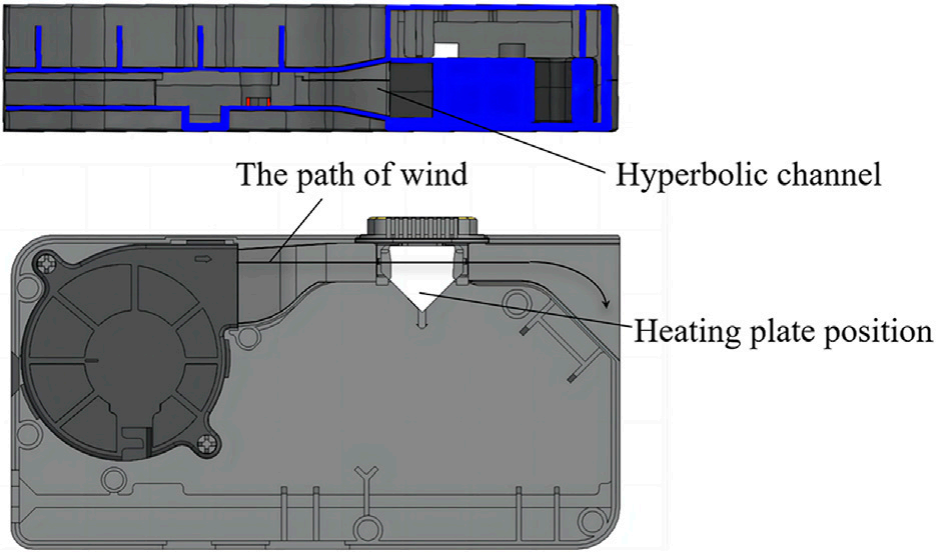
Schematic diagram of the hyperbolic wind cooling unit.

The cooling unit is made up of a high-speed magnetic levitation cooling fan and hyperbolic channel. Here, hyperbolic wind cooling is used to increase cooling efficiency (Figure 7). Structure, process, and design optimizations of the heating and cooling units enabled a heating rate of 15°C/s, reducing a 1.5–2 h procedure to under 30 min.

**FIGURE 8:**
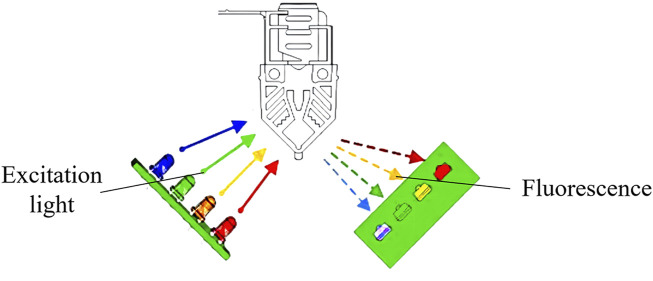
Schematic diagram of the fixed light path fluorescence detection unit.

The fixed light path fluorescence detection unit consists of an excitation light source and a light detector. The excitation light source consists of four independent excitation channels, and the light detector contains four independent detection channels. The excitation channels of the light source use light-emitting diodes, which is reflected through filters into the PCR reaction cups. Fluorophores in the PCR reaction cup emits fluorescence light that is reflected through filters into the light detectors, which use PIN photodiodes. Optimal signal-to-noise ratio is achieved through a 90° fluorescence detection angle between the excitation light source and the light detector (Figure 8). The four independent excitation channels can simultaneously detect four fluorescent dyes with different wavelengths, enabling simultaneous identification of four targets. The fixed light path fluorescence detection unit is manufactured through injection molding, with the spectroscopy and optical path collimation components for fluorescence detection manufactured through molds and no moving parts in the entire fixed light path fluorescence detection unit. This design allows the device to be moved, tilted, or placed vertically or at any other angle, enhancing its usability in a wide range of scenarios.

The lyophilization reagent unit (Zhuhai Livzon Diagnostics Inc.) consists of lyophilization reagents based on the PCR fluorescence probe design for COVID-19, influenza A, and influenza B triple testing. Primer probes in the reagent are designed to target the SARS-CoV-2 ORF1ab and N genes as well as specific IAV and IBV conserved nucleic acid sequences. Real-time fluorescent quantitative PCR enables the *in vitro* qualitative detection of SARS-CoV-2, IAV, and IBV RNA through changes in fluorescence signals. Endogenous internal standards are used to monitor sample extraction, enrichment, and PCR reaction to avoid false negative results. Simultaneously, uracil-N-glycosylase is used as an anticontaminant. This enzyme degrades uracil amplification products to prevent false positives.

### 2.3 Smartphone data processing module

The smartphone data processing module consists of the Bluetooth serial port transfer unit and a smartphone application (app) unit. Fluorescence signals received by the light detector are converted to digital signals through an analog-to-digital converter. Commands from the communication protocol connects the nucleic acid extraction and enrichment module and ultrafast thermal cycling PCR module to the smartphone data processing module. After a connection is established, the nucleic acid extraction and enrichment module and ultrafast thermal cycling PCR module sends data to the smartphone data processing module through the Bluetooth serial port using the corresponding communication protocol.

To ensure the data stability and accuracy between modules, the smartphone data processing module is used to establish a communication with the PCR module through an identification code, which allows the transmission of program instruction packets to the PCR module. After receiving the response confirmation data packet (ACK data packet) feedback from the PCR module, subsequent program instruction packets are sent. To avoid data transmission delays and unstable signals, intermediate communication units, such as routers, switches, and other communication equipment, may be added.

**FIGURE 9:**
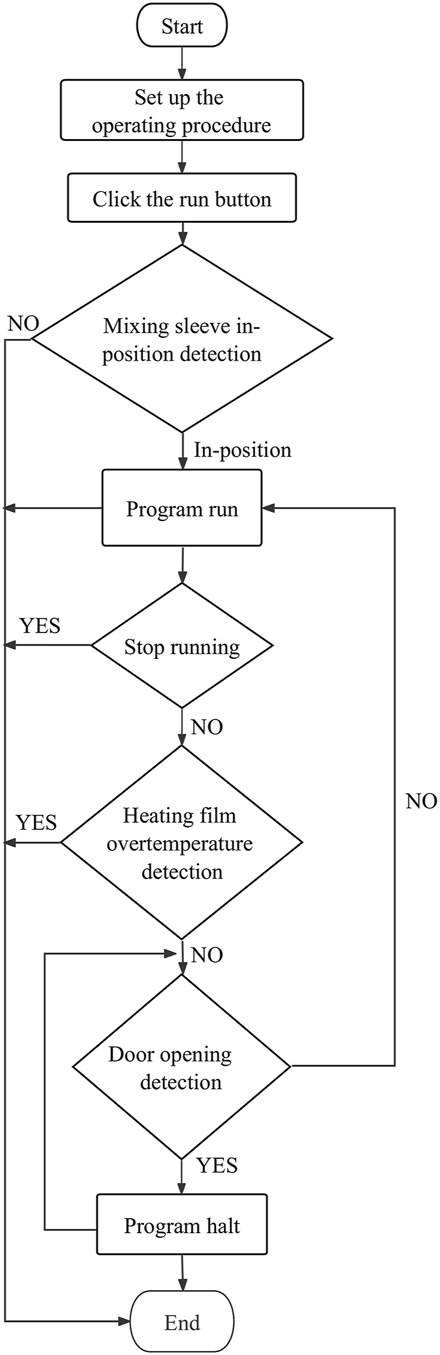
Sample extraction and enrichment module workflow (left).

**FIGURE 10:**
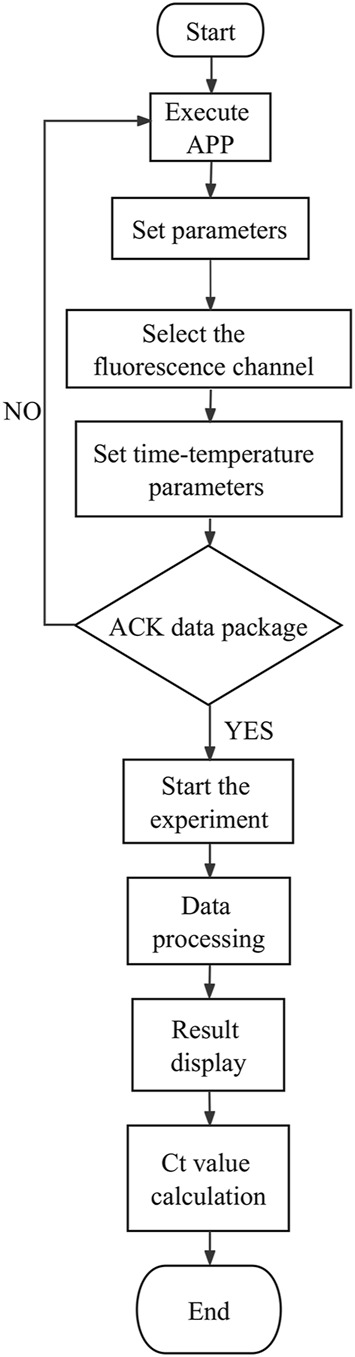
Ultrafast thermal cycling PCR module work flow (right).

The data processing module realizes the wireless connection between the smartphone and the sample extraction and enrichment module and the ultrafast thermal cycle PCR module for near-field communication. The module sends program instruction package through a self-developed app to activate nucleic acid extraction, ultrafast thermal cycling, and real-time fluorescence detection. It receives feedback from the execution results, which is modulated, calculated, and processed by the smartphone’s central processing unit. The detection results are displayed through images and text on the phone screen. The app was developed on Android Studio programmed in Java. It is based on Bluetooth Low Energy central. The app can interact with the sample extraction and enrichment module and ultrafast thermal cycling PCR module by setting parameters and controlling their initiation and termination, the operation procedures of which are shown in Figures 9, 10. The application allows for the convenient and rapid real-time observation of experiment progress, generation of temperature and amplification curves, and acquisition of experimental data and various plots based on user needs. The app is graphical, can processes data rapidly, and is highly accurate.

## 3 Experimental materials and methods

### 3.1 Reference standards

SARS-CoV-2 nucleic acid national reference standard 370,099-202001(National Institutes for Food and Drug Control, China), SARS-CoV-2 nucleic acid validation reference standard 202306011 (Guangzhou BDS Biological Technology Co., Ltd., China), and second-generation influenza A/B nucleic acid test reagent national reference standard 370,007-202202 (National Institutes for Food and Drug Control, China) were used as reference standards.

### 3.2 Experimental methods

Performance verification of the COVID-19, influenza A, and influenza B triple POCT system for testing SARS-CoV-2, IAV, and IBV was made using reference standards and clinical samples. The sample extraction and enrichment module was used to extract sample RNA, which was then added to the lyophilization reagent and amplified on the ultrafast thermal cycling PCR module. The reaction conditions are 50°C for 180 s, 95°C for 5 s, (95°C for 1 s, 60°C for 20 s) for 45 cycles. At 60°C for 20 s, the fluorescence detection channels were set as VIC (IAV target gene), CY5 (IBV target gene), FAM (SARS-CoV-2 target gene), and ROX (internal reference gene). The positive threshold value for the detection of SARS-CoV-2, IAV, and IBV in lyophilization reagent is (cycle threshold) Ct ≤ 40. GraphPad Prism nine was used for linear regression analysis to obtain the regression formula and linear correlation coefficient R.

## 4 Results

### 4.1 Evaluating the performance of SARS-CoV-2 detection by the COVID-19, influenza A, and influenza B triple POCT system

#### 4.1.1 Limit of detection and linear range

**FIGURE 11:**
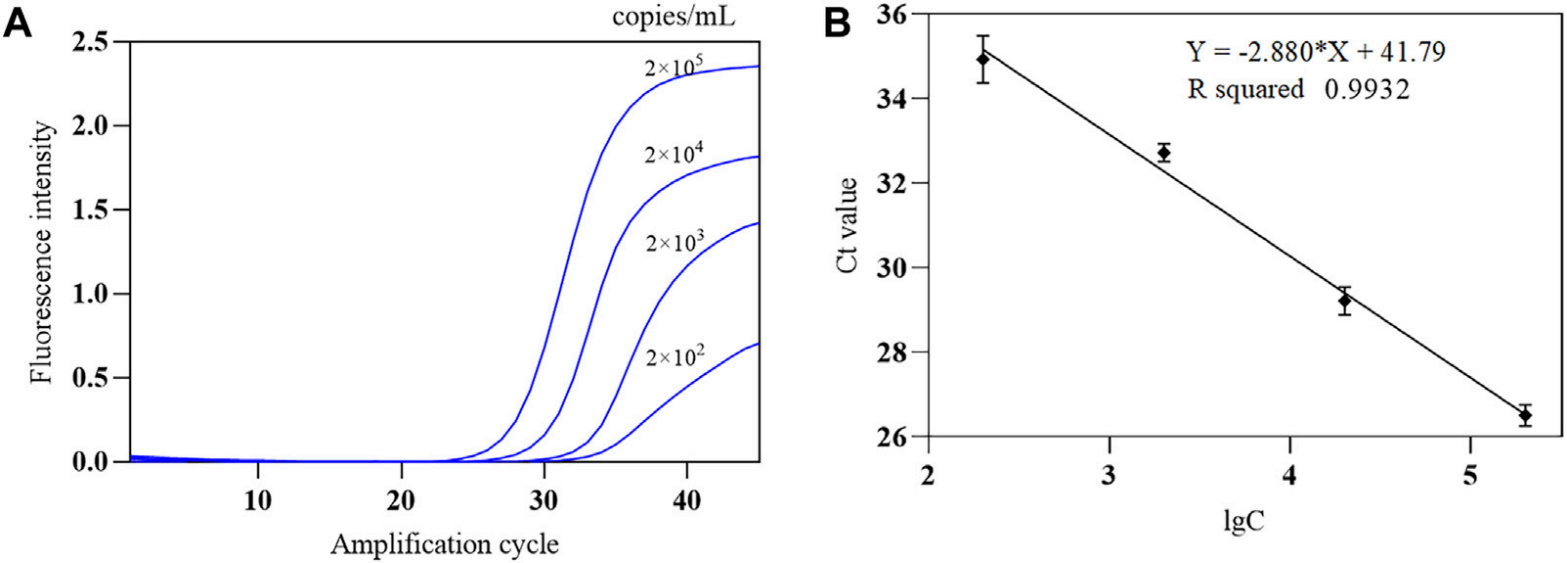
The amplification curve and standard curve of SARS-CoV-2 sensitivity reference standard detected by the COVID-19, influenza A, and influenza B triple POCT system **(A)**. Amplification curves of the triple POCT system for SARS-CoV-2 at various concentrations; **(B)**. Standard curve of SARS-CoV-2 detected by the triple POCT system.

The SARS-CoV-2 nucleic acid test reagent national sensitivity reference standard S with a concentration of 3 × 10^5^ copies/mL was used for testing. The reference standard was diluted with nuclease-free water to 2 × 10^5^ copies/mL, followed by 1:10 serial dilution to obtain samples at four different concentrations (2 × 10^2^ to 2 × 10^5^ copies/mL). The COVID-19, influenza A, and influenza B triple POCT system was used to test each concentration five times, and the lowest concentration level with 100% detectability was determined as the minimum detection limit. Results showed that the lower limit of detection of this system for SARS-CoV-2 was 200 copies/mL. A standard curve was generated from the mean Ct of the amplification curve against the logarithm of the concentration (lgC) of reference standard S on the x-axis. Figure 11A shows the amplification curves for various SARS-CoV-2 concentrations. Figure 11B shows the standard curve for SARS-CoV-2 for the triple POCT system. The linear range for SARS-CoV-2 detection by the system is 2 × 10^2^ to 2 × 10^5^ copies/mL.

#### 4.1.2 Conformity rate of positive and negative reference standards

**FIGURE 12:**
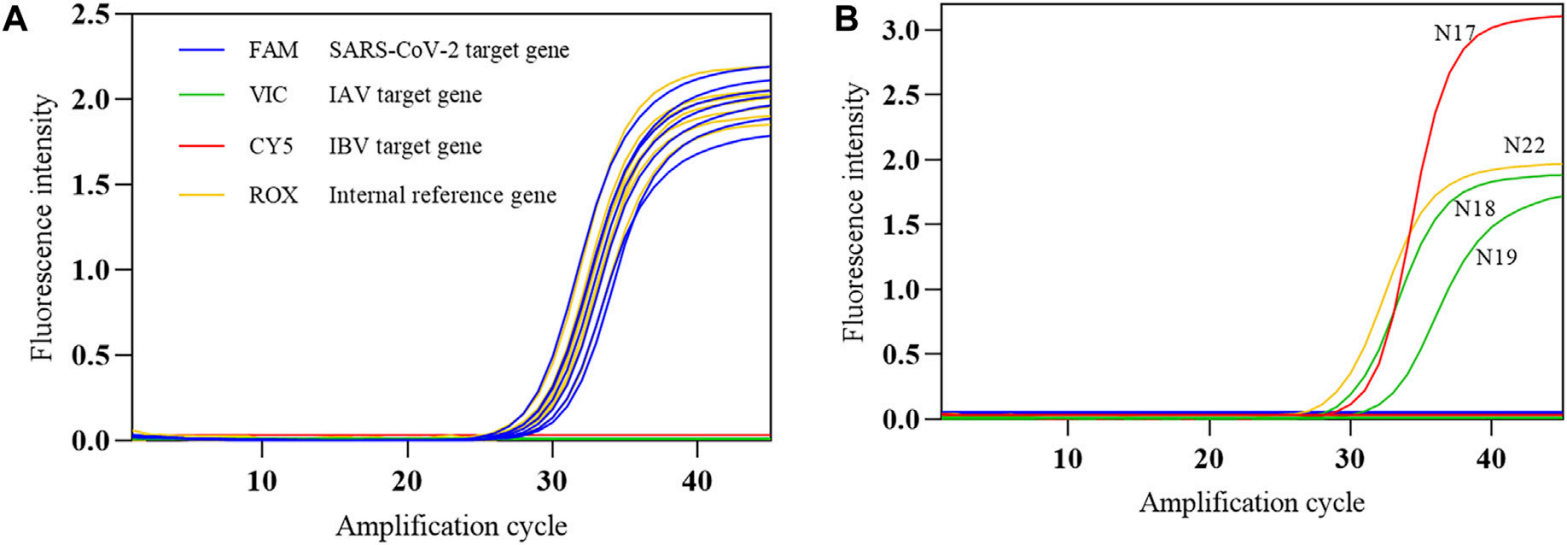
The amplification curves of SARS-CoV-2 reference standards detected by the COVID-19, influenza A, and influenza B triple POCT **(A)**. Amplification curve of positive reference standards; **(B)**. Amplification curve of negative reference standards.

The COVID-19, influenza A, and influenza B triple POCT system was used to test the SARS-CoV-2 nucleic acid national positive reference standard (P1–P7) and negative reference standard (N1–N22). The conformity rate (+/+) of seven positive reference standards was 100%, and the conformity rate of 22 negative reference standards was 100% (Figures 12A, B). In Figure 12B, N17 is positive IBV (Victoria); N18 and N19 are influenza A H1N1(2009) and influenza A H3N2 virus positive samples, respectively. N22 is a negative simulation pharyngeal swab sample containing human-derived cells. These results show that this system can accurately identify positive samples. We were also able to determine that the triple POCT does not cross-react with coronaviruses HKU1, OC43, NL63, 229E, MERS; influenza virus; parainfluenza virus; respiratory syncytial virus; adenovirus; *Streptococcus pneumoniae*; and *Haemophilus influenzae*, showing that the system has high specificity.

#### 4.1.3 Precision test

**FIGURE 13:**
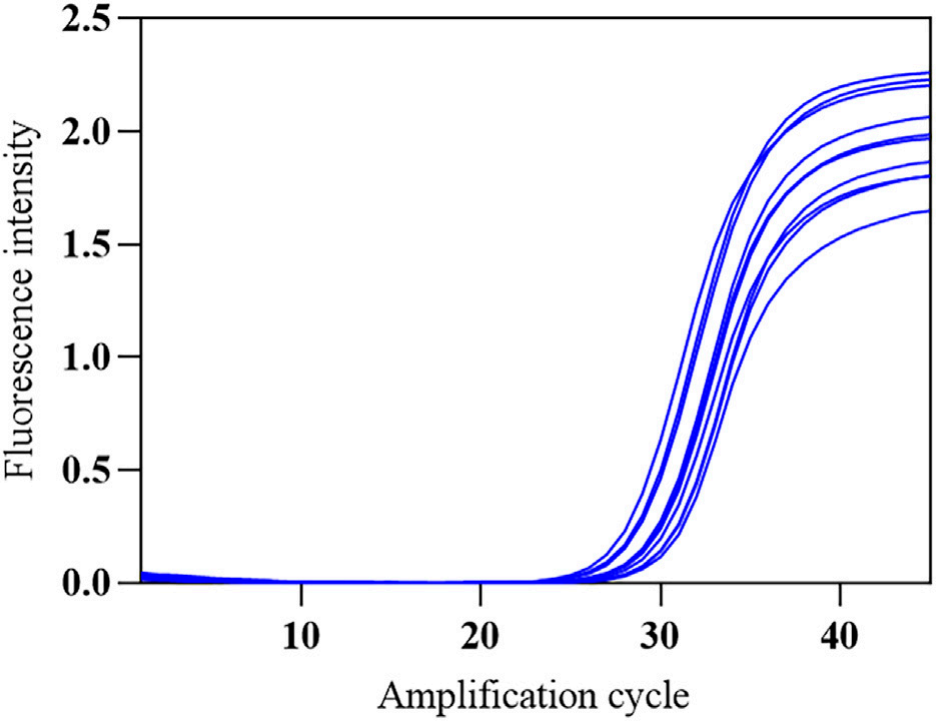
Amplification curves of SARS-CoV-2 precision reference standards.

The SARS-CoV-2 nucleic acid test reagent national precision reference standard R was selected and diluted 1:20 with nuclease-free water. The standard was tested ten times on the COVID-19, influenza A, and influenza B triple POCT system to obtain the corresponding mean Ct and its standard deviation and coefficient of variation. Figure 13 shows the amplification curves obtained from the ten replicate tests. Positive results were obtained for all ten tests, with a coefficient of variation of 3.76% for the Ct. Here, the triple POCT reliably and reproducibly detects SARS-CoV-2.

#### 4.1.4 Interference tolerance

**FIGURE 14:**
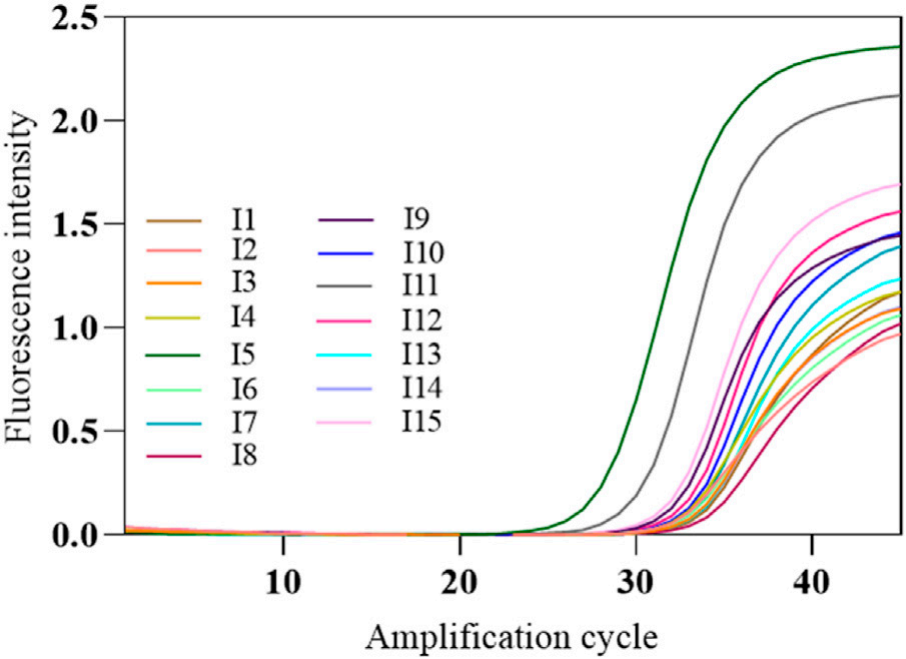
Amplification curves of SARS-CoV-2-interfering substance reference standards. The amplification curve of SARS-CoV-2 target gene was shown in the figure. The interference reference standards I1–I14 respectively include 2-g/L hemoglobin, 6-g/dL albumin, 100-μg/mL ribavirin + azithromycin, 0.1-mg/mL bilirubin, 3.2-g/dL triglycerides, 18-g/L total lgG, 18-g/L total lgM, >1:50 antinuclear antibody, 100-μg/mL oseltamivir, 100-μg/mL azithromycin, 100-μg/mL ceftriaxone, 100-μg/mL tobramycin, 200-μg/mL histamine hydrochloride, 60-μg/mL sodium chloride, with I15 being a normal sample control without interfering substances.

The COVID-19, influenza A, and influenza B triple POCT system’s tolerance for interference was tested with the SARS-CoV-2 nucleic acid test performance interference reference standards I1–I15, which validates the system’s capacity to resist interference from potential endogenous/exogenous substances such as blood, antibiotics, antivirals, and antiallergy medications. The interference reference standards I1–I14 respectively include 2-g/L hemoglobin, 6-g/dL albumin, 100-μg/mL ribavirin + azithromycin, 0.1-mg/mL bilirubin, 3.2-g/dL triglycerides, 18-g/L total lgG, 18-g/L total lgM, >1:50 antinuclear antibody, 100-μg/mL oseltamivir, 100-μg/mL azithromycin, 100-μg/mL ceftriaxone, 100-μg/mL tobramycin, 200-μg/mL histamine hydrochloride, 60-μg/mL sodium chloride, with I15 being a normal sample control without interfering substances. The results showed that PCR successfully amplified SARS-CoV-2 nucleic acids and internal standard in the presence of interfering substances such as albumin, bilirubin, and triglycerides (Figure 14).

### 4.2 Evaluating the performance of IAV and IBV detection by the COVID-19, influenza A, and influenza B triple POCT system

#### 4.2.1 Limit of detection and linear range

The second-generation influenza A/influenza B virus nucleic acid national limit of detection reference standard L3 (influenza A H1N1) with a concentration of 5.5 × 10^6^ copies/mL was used to test the triple POCT system. The standard was diluted with nuclease-free water to 2 × 10^6^ copies/mL, which was then serially diluted to 1:10 to obtain five different concentrations (2 × 10^2^ to 2 × 10^6^ copies/mL). The second-generation influenza A/influenza B virus nucleic acid national limit of detection reference standard L1 (B/Victoria) with a concentration of 1.4 × 10^7^ copies/mL was used for testing. RNase/DNase-free deionized water was used to dilute the standard to 2 × 10^6^ copies/mL, followed by 1:10 serial dilution to obtain five different concentrations (2 × 10^2^ to 2 × 10^6^ copies/mL).

**FIGURE 15:**
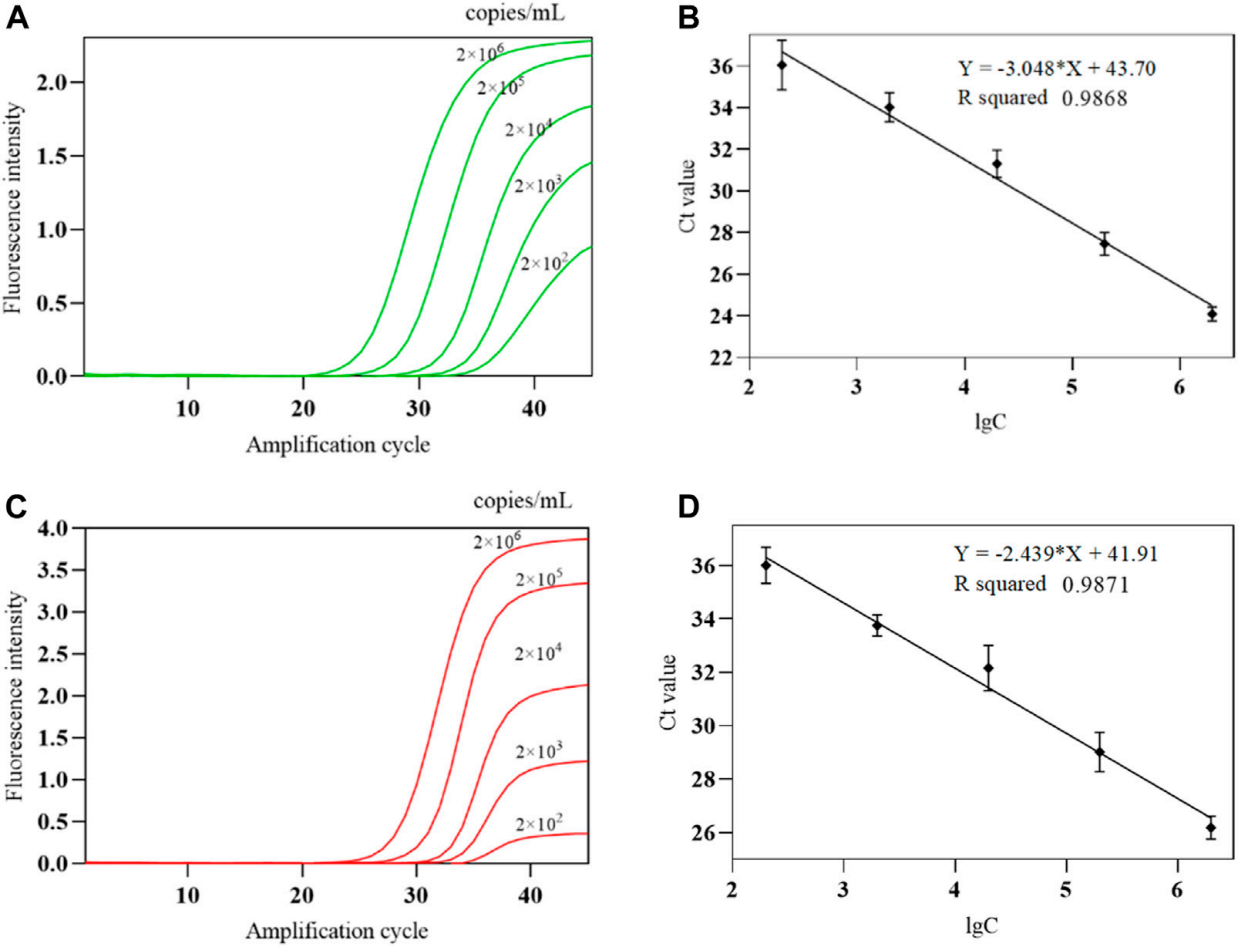
The amplification curve and standard curve of IAV、IBV detected by the COVID-19, influenza A, and influenza B triple POCT system **(A)**. Amplification curves of IAV at various concentrations; **(B)**. Standard curve of IAV detected by the triple POCT system; **(C)**. Amplification curves of IBV at various concentrations; **(D)**. Standard curve of IBV detected by the triple POCT system.

The COVID-19, influenza A, and influenza B triple POCT system was used to test each concentration five times, and the 100% detected lowest concentration was used as the lower limit of detection. The experimental results showed that the lower limit of detection of this system for IAV and IBV is 200 copies/mL. A standard curve was generated by plotting the mean Ct of the amplification curve against the lgC of sensitivity reference standard S as the x-axis. Figures 15A, C show the amplification curves for various IAV and IBV concentrations, respectively. Figures 15B, D show the standard curves for IAV and IBV from the system. The linear range for IAV and IBV detection by the system is 2 × 10^2^ to 2 × 10^6^ copies/mL.

#### 4.2.2 Conformity rate of positive and negative reference standards

The COVID-19, influenza A, and influenza B triple POCT system was used to test the second-generation IAV/IBV nucleic acid national positive reference standards (P1–P10) and negative reference standards (N1–N10). The conformity rate (+/+) of ten positive reference standards was 100%, and the conformity rate of ten negative reference standards was also 100%. Here, the results demonstrate that this system can accurately identify positive samples, with no cross-reactivity with *Neisseria meningitidis*, *Mycoplasma pneumoniae*, *Staphylococcus aureus*, *Streptococcus pneumoniae*, rhinovirus, coronavirus 229E, adenovirus, or other respiratory pathogens with high specificity.

#### 4.2.3 Precision test

The IAV/IBV nucleic acid test reagent national repeatability reference standard R1 was selected and diluted 1:20 with nuclease-free water. The standard was tested for ten times on the COVID-19, influenza A, and influenza B triple POCT system to obtain the corresponding mean Ct, as well as its standard deviation and the coefficient of variation. All ten tests results were positive for influenza B and negative for influenza A. The coefficient of variation for Ct was 2.96%. The same test method was used for R2, with all ten test results positive for influenza A and negative for influenza B. The coefficient of variation for Ct was 4.34%. Our results show that this system reliably detects IAV/IBV, and it is reproducible.

### 4.3 Clinical application evaluation of the COVID-19, influenza A, and influenza B triple POCT system

The COVID-19, influenza A, and influenza B triple POCT system was used to test 125 clinical samples to determine the accuracy of this system for testing clinical samples. The clinical samples consisted of pharyngeal swabs collected between 2019 and 2023 by the Center for Disease Prevention and Control of the Southern Theatre Command. The center staff screened positive and negative results using PCR. Clinical samples were stored in 1–3 mL of preservation solution at −80 °C. The samples were grouped as follows: (1) Confirmed IAV positive group: 32 patients; (2) Confirmed IBV positive group: 35 patients; (3) Confirmed SARS-CoV-2 positive group: 43 patients; (4) IAV, IBV, and SARS-CoV-2 excluded group: 15 patients (Table 1). The test results showed that this system has high sensitivity and specificity for testing clinical samples with no interference between the detection of IAV, IBV, and SARS-CoV-2.

**TABLE 1:** Clinical sample results tested by the triple POCT system.

Sample	Test	Result		Sensitivity	Specificity (%)	Mutual interference
		Positive (n)	Negative (n)			
Confirmed IAV positive (n = 32)	1.IAV	31	1	96.88%	100	No interference
	2.IBV	0	32			
	3.SARS-CoV-2	0	32			
Confirmed IBV positive (n = 35)	1.IAV	0	35	97.14%	100	No interference
	2.IBV	34	1			
	3.SARS-CoV-2	0	35			
Confirmed SARS-CoV-2 positive (n = 43)	1.IAV	0	43	95.35%	100	No interference
	2.IBV	0	43			
	3.SARS-CoV-2	41	2			
IAV, IBV, SARS-CoV-2 excluded group (n = 15)	1.IAV	0	15	-	100	No interference
	2.IBV	0	15			
	3.SARS-CoV-2	0	15			

## 5 Conclusion

In this study, we designed and manufactured the COVID-19, influenza A, and influenza B triple POCT system, which allowed for the concurrent detection of SARS-CoV-2, IAV, and IBV through nucleic acid testing within 30 min. This test is highly automated, universal, “smart”, and cloud-based. Various technical indicators assessed in this study (lower limit of detection, sensitivity, specificity, and stability) have reached or were close to those of fluorescence quantitative PCR. Hence, the triple POCT system may be suitable for hospital outpatient or emergency department for onsite testing as a screening device in airplanes, ports, and customs. The triple POCT system can also be used for mobile testing on automobiles, ships, and airplanes. Advances in the research on flexible pipetting technology may facilitate the eventual design of a fully automated, closed “sample in, result out”, advanced POCT system.

## Data Availability

The original contributions presented in the study are included in the article/Supplementary Material, further inquiries can be directed to the corresponding authors.
