# Supplementary figures and images for: Design and application of a point-of-care testing system for triple detection of SARS-CoV-2, influenza A, and influenza B
Source: Front Bioeng Biotechnol. 2024 Apr 17;12:1378709. doi: 10.3389/fbioe.2024.1378709 (PMC11061352; doi:10.3389/fbioe.2024.1378709)

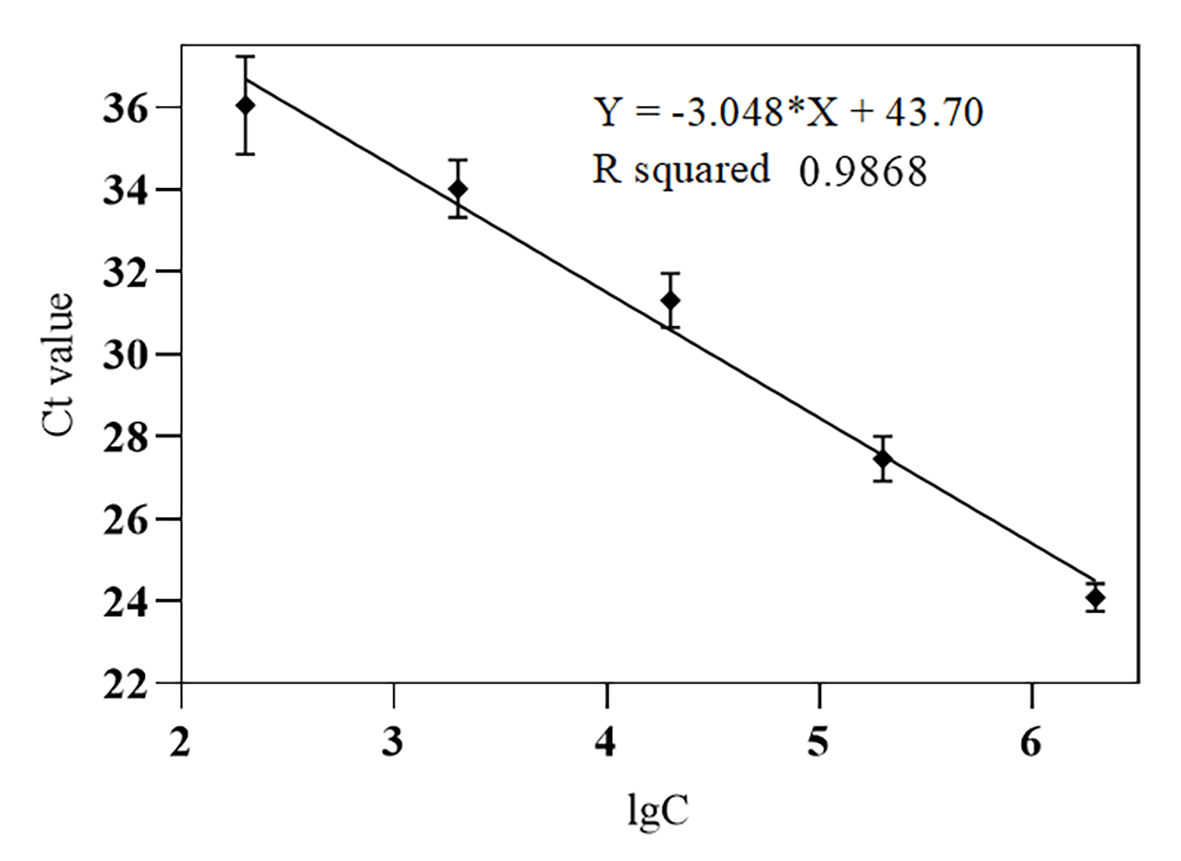

Supplement: Supplementary file 1 [file Image3.TIF]

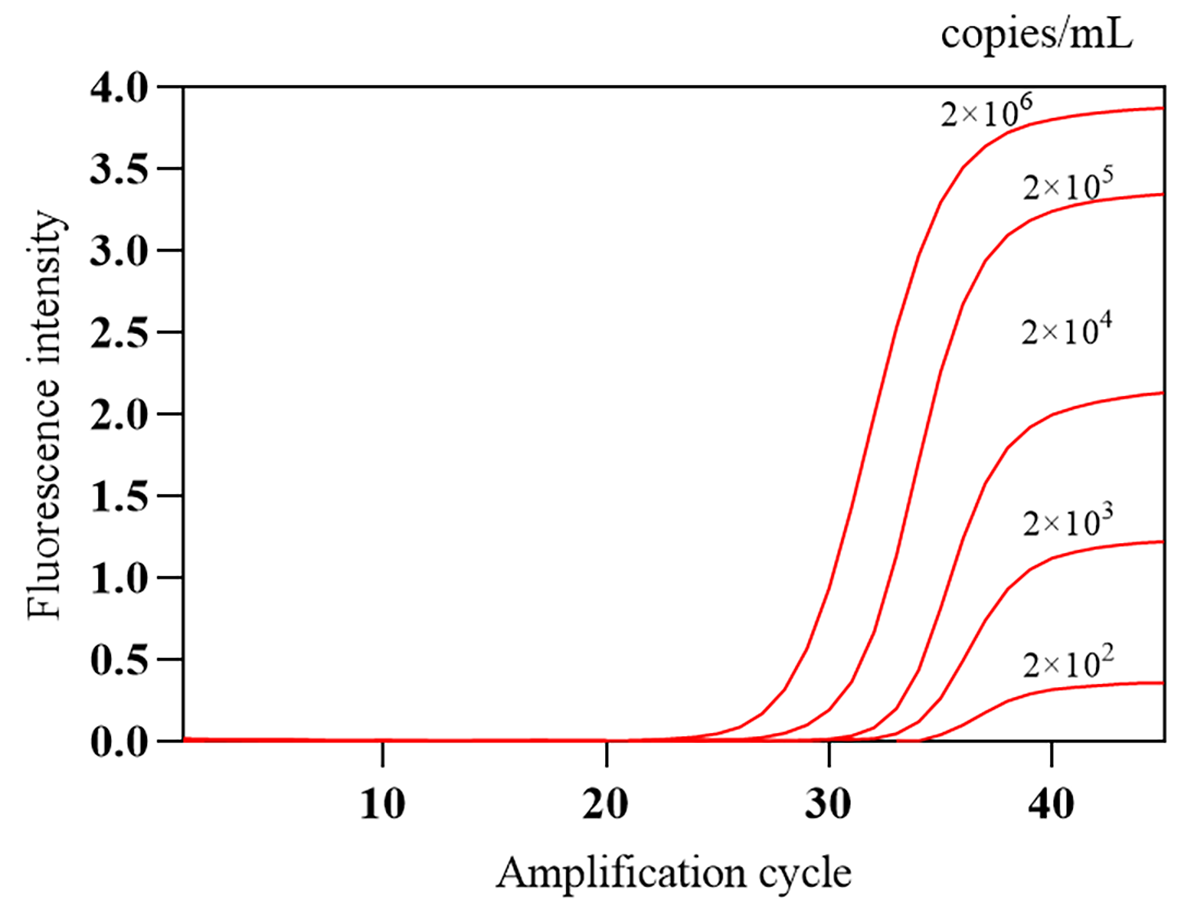

Supplement: Supplementary file 2 [file Image4.TIF]

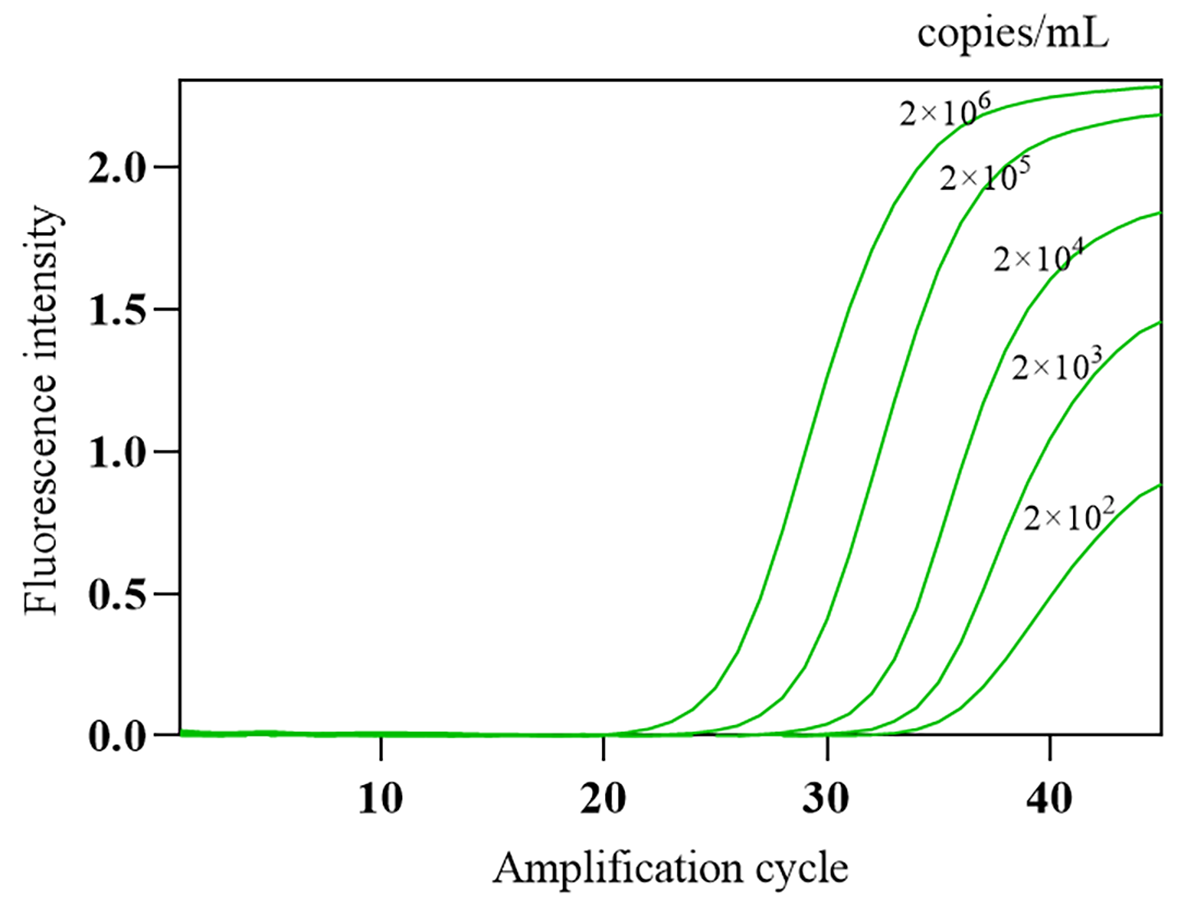

Supplement: Supplementary file 3 [file Image2.TIF]

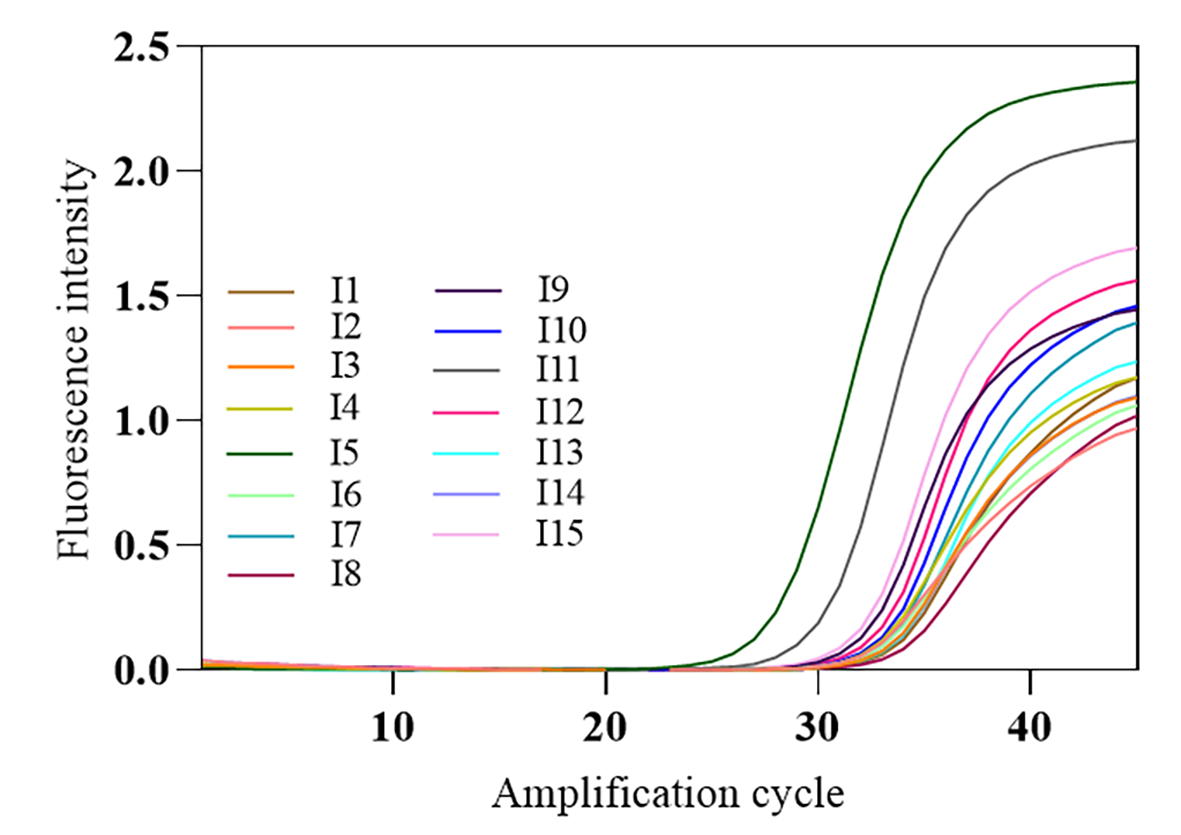

Supplement: Supplementary file 4 [file Image1.TIF]

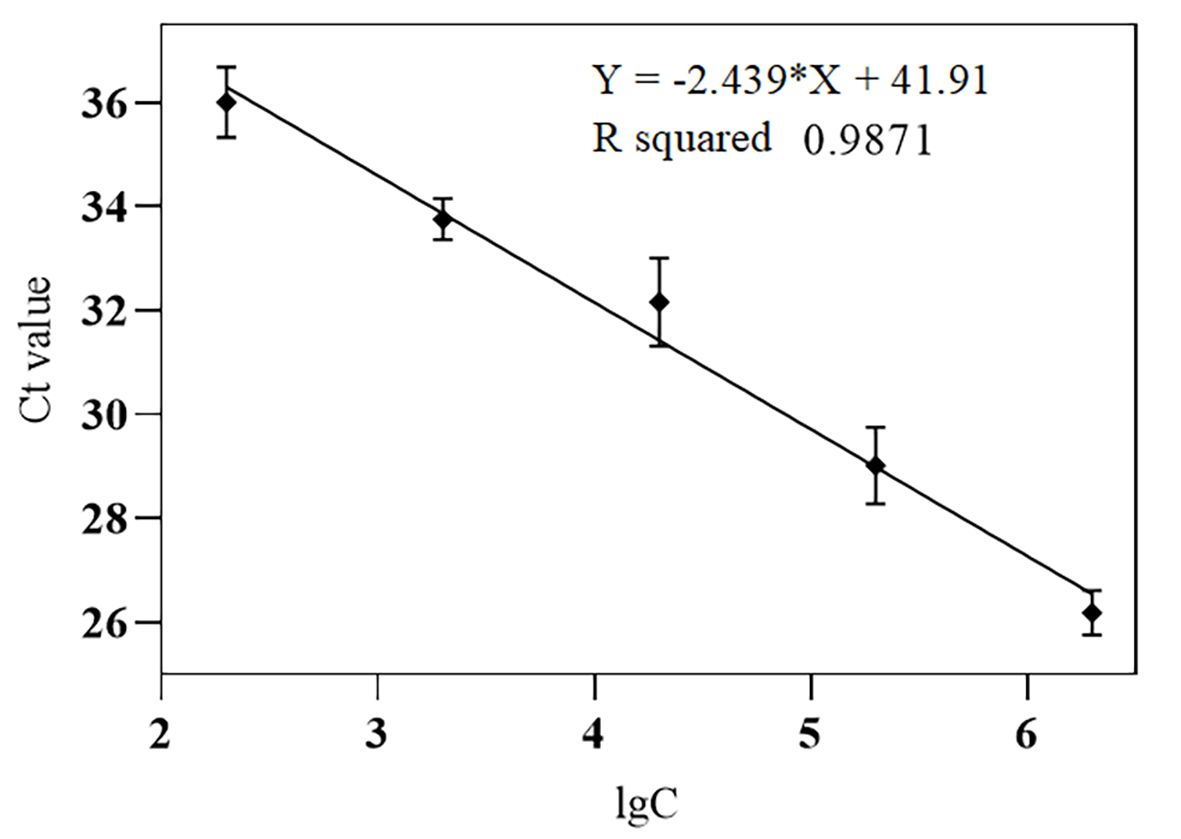

Supplement: Supplementary file 5 [file Image5.TIF]
